# Primary progressive aphasia and motor neuron disease: A review

**DOI:** 10.3389/fnagi.2022.1003792

**Published:** 2022-09-08

**Authors:** Edoardo Nicolò Aiello, Sarah Feroldi, Giulia De Luca, Lucilla Guidotti, Eleonora Arrigoni, Ildebrando Appollonio, Federica Solca, Laura Carelli, Barbara Poletti, Federico Verde, Vincenzo Silani, Nicola Ticozzi

**Affiliations:** ^1^Department of Neurology and Laboratory of Neuroscience, IRCCS Istituto Auxologico Italiano, Milan, Italy; ^2^Ph.D. Program in Neuroscience, School of Medicine and Surgery, University of Milano-Bicocca, Monza, Italy; ^3^Department of Brain and Behavioral Sciences, University of Pavia, Pavia, Italy; ^4^Department of Psychology, University of Milano-Bicocca, Milan, Italy; ^5^Neurology Section, School of Medicine and Surgery, University of Milano-Bicocca, Monza, Italy; ^6^Department of Pathophysiology and Transplantation, “Dino Ferrari” Center, Università degli Studi di Milano, Milan, Italy

**Keywords:** primary progressive aphasia, motor neuron disease, frontotemporal degeneration, amyotrophic lateral sclerosis, language

## Abstract

**Background:**

This study aims at reviewing, within the framework of motor neuron disease-frontotemporal degeneration (MND-FTD)-*spectrum* disorders, evidence on the co-occurrence between primary progressive aphasia (PPA) and MND in order to profile such a complex at pathological, genetic and clinical levels.

**Methods:**

This review was pre-registered (osf.io/ds8m4) and performed in accordance with the 2020 PRISMA guidelines. Case reports/series and group studies were included if addressing (1) progressive non-fluent aphasia (PNFA) or semantic dementia (SD) with MND or (2) MND patients with co-morbid PNFA/SD.

**Results:**

Out of 546 initial records, 56 studies were included. As to case reports/series (*N* = 35), which included 61 PPA-MND patients, the following findings yielded: (1) PNFA is more frequent than SD in PPA-MND; (2) in PPA-MND, the most prevalent motor phenotypes are amyotrophic lateral sclerosis and predominant-upper MND, with bulbar involvement being ubiquitous; (3) extrapyramidal features are moderately frequent in PPA-MND; (4) PPA-MND patients usually display frontotemporal, left-greater-than-right involvement; (5) TDP-43-B is the typical pathological substrate of PPA-MND; (6) *TBK1* mutations represent the most frequent genetic risk factors for PPA-MND.

As to group studies, including 121 patients, proportional meta-analytic procedures revealed that: (1) the lifetime prevalence of MND in PPA is 6%; (2) PPA occurs in 19% of patients with co-morbid MND and FTD; (3) MND is more frequent in PNFA (10%) than in SD patients (3%).

**Discussion:**

Insights herewith delivered into the clinical, neuropathological and genetic features of PPA-MND patients prompt further investigations aimed at improving clinical practice within the MND-FTD *spectrum*.

## Introduction

Due to the pathophysiological and genetic grounds shared by motor neuron diseases (MNDs) and frontotemporal degenerations (FTDs), these two disorders belong to the same nosological entity, i.e., the “MND-FTD *spectrum*” (Burrell et al., 2016). FTD-like cognitive and behavioral dysfunctions indeed occur in up to 50% of MND patients (Strong et al., 2017), 5–15% of whom showing a full-blown FTD (Montuschi et al., 2015). However, such a *spectrum* can be also “read backward” – i.e., as “FTD-MND” (Strong et al., 2017): evidence of upper (UMN) and/or lower motor neuron (LMN) dysfunction can be in fact detected in up to 30% of FTD patients (Burrell et al., 2011; Cerami et al., 2015; Gromicho et al., 2021).

Among FTDs, the behavioural variant (bvFTD) is the most prevalent phenotype associated with MNDs (Saxon et al., 2017a,b; Wagner et al., 2021): the co-occurrence of MND or UMN/LMN dysfunction and bvFTD has been indeed thoroughly explored (Ahmed et al., 2021). By contrast, little is still known about the pathology, genetics and clinical features of co-occurring primary progressive aphasia (PPA) and MND (Ulugut et al., 2021). In the light of the diagnostic (Strong et al., 2017; Suárez-González et al., 2021) and prognostic relevance (Garcin et al., 2009; Ahmed et al., 2020; De La Sablonnière et al., 2021) of language disorders in MND patients and of motor neuron (MN) dysfunction in PPA patients, the present study aims at systematically reviewing evidence on the co-occurrence between PPA and MND (hereafter referred to as “PPA-MND”).

## Methods

This review was pre-registered (OSF Registries: osf.io/ds8m4) and performed in accordance with the 2020 revision of Preferred Reporting Items for Systematic Reviews and Meta-Analyses guidelines (PRISMA) (Page et al., 2021).

### Search

Records were searched for in Scopus and PubMed on August 8th, 2021 through the following string: (“motor neuron disease” OR “amyotrophic lateral sclerosis”) AND (“primary progressive aphasia” OR “progressive aphasia” OR “progressive non-fluent aphasia” OR “progressive non-fluent aphasia” OR “semantic dementia” OR “semantic variant” OR “agrammatic variant” OR “non-fluent variant” OR “non-fluent variant”). The string did not referred to logopenic-variant PPA (lvPPA), as being commonly underpinned by a neuropathology different from frontotemporal lobar degeneration (FTLD), i.e., that of Alzheimer’s disease (AD) (Roytman and Chiang, 2022).

Fields of search were the title, abstract and possibly key words. No date limit was set. Only peer-reviewed articles written in English, Italian, German, French or Spanish were considered. Records not indexed within online databases were not searched for. Further potentially relevant contributions were retrieved from the reference lists of included ones.

### Inclusion and exclusion criteria

For a study to be included, it had to address either (1) progressive non-fluent aphasia (PNFA) or semantic dementia (SD) patients with MND or clinical/electromyographic evidence of MN dysfunction or (2) MND patients with co-morbid PNFA/SD.

Either Neary et al.’s (1998) or Gorno-Tempini et al.’s (2011) *criteria* were addressed for PNFA and SD diagnoses.

If neither of the two nosographic systems were referred to, a clinical diagnosis of PPA and/or a cluster of language deficits consistent with a progressive, either fluent or non-fluent aphasic syndrome was deemed as satisfactory for inclusion, based on a joint decision of two Authors expert in aphasiology (E.N.A. and S.F.).

Studies describing anarthric patients whose language was assessable only through writing were included only if an explicit mention to aphasia was made (Aiello et al., 2021).

Both case report/series and group studies were addressed as eligible. Abstracts, reviews, meta-analyses, research protocols and opinion papers were excluded.

### Data collection and synthesis

Screening and eligibility checks were performed by two independent Authors (E.N.A. and S.F.), with a third independent Author supervising and solving disagreements (I.A.). Decisions during screening and eligibility stages were performed *via* Rayyan^1^. Data were extracted by four Authors (S.F., G.D.L., L.G., and E.A.) and independently checked by a third Author (E.N.A.).

The following outcomes were extracted: (1) age and sex; (2) PPA phenotype; (3) MND phenotype – according to Chiò et al.’s (2011) classification – or presence of MN dysfunction; (4) neuropsychological vs. motor onset; (5) survival; (6) clinical course, i.e., whether PPA or MND occurred first, and timespan between their occurrence; (7) bulbar signs at MND onset and their lifetime prevalence; (8) early bulbar involvement in “PPA-first” patients; (9) presence and type of extrapyramidal involvement; (10) cortical neuroradiological findings; (11) neuropathological findings; (12) genetics.

A qualitative synthesis of extracted data was performed by inserting them within pilot-tested tables. Additionally, by addressing studies including ≥30 patients, possibly eligible for prevalence/incidence estimation, a proportional meta-analytic procedure *via* the R package *metafor* (Viechtbauer, 2010) was performed, by addressing a random-effect model with *I*^2^ as the heterogeneity measure.

## Results

Study selection process is shown in Supplementary Figure 1. Out of 546 initial unique records, 56 studies were included, of which 36 were case reports/series (Table 1; *N* = 61 patients) and 20 group studies (Table 2; *N* = 121 patients).

**TABLE 1:** Case reports and series.

Authors, year	Sex, age (year)	Genetics	MND pheno type	PPA pheno type	NPs vs. motor onset	Side of motor onset	Bulbar signs at any time	Bulbar signs at MND onset	Bulbar signs concurrent to PPA onset	EPD	Survival	Main cortical imaging findings	Main histological findings	Clinical course, latency
Kirshner et al., 1987	M, 61	n.r.	n.s.	n.s.	Language	n.r.	−	n.a.	n.a.	−	5 year	Widespread	Aspecific (ND)	PPA → MND; 48 months
Doran et al., 1995	F, 65	n.r.	n.s.	n.s.	Language	n.r.	+	−	−	−	n.r.	Morph.: T (L)	AD	PPA → MND
	F, 64	n.r.	n.s.	n.s.	Behavioral	n.r.	+	+	+	−	57 months	Morph.: widespread	LBD	PPA + MND
	M, 72	n.r.	n.s.	n.s.	n.r.	n.r.	+	n.a.	n.a.	−	30 months	Morph.: widespread; F-P; T (L) funct.: T-P (L)	n.r.	n.r.
	M, 59	n.r.	n.s.	n.s.	Language + motor	n.r.	+	−	−	−	30 months	Morph.: − funct.: widespread; T-P (L)	n.r.	PPA + MND
	F, 43	n.r.	n.s.	n.s.	Language + motor	n.r.	+	+	+	−	n.a.	Morph.: widespread; T (L) funct.: T-P (L)	n.r.	PPA + MND
Mimura et al., 1998	F, 56	n.r.	n.s.	PNFA	Language	n.r.	+	−	−	−	27 months	Morph.: F-T	UP	PPA → MND 11 months
	M, 49	n.r.	n.s.	PNFA	Language-behavioral	n.r.	+	+	−	−	34 months	Morph.: widespread	UP	PPA → MND 29 months
Tsuchiya et al., 2000	F, 74	n.r.	ALS	PNFA	Language + motor	n.r.	+	+	+	−	10 months	Morph.: T (L)	UP	PPA + MND
Bak et al., 2001	M, 49	n.r.	ALS	PNFA	Language- behavioral	n.r.	+	−	−	−	36 months	Morph.: − funct.: F	UP	PPA → MND 6 months
	M, 67	n.r.	ALS	PNFA	Language	n.r.	+	−	−	−	18 months	morph.: T (L) funct.: P	UP	PPA → MND 2 months
	M, 49	n.r.	ALS	PNFA	Behavioral + motor	n.r.	+	+	n.a.	−	22 months	Morph.: F funct.: F	Aspecific (ND)	PPA + MND
	M, 64	n.r.	ALS	PNFA	Language + motor	n.r.	+	+	n.a.	−	≈24 months	Morph.: T (L)	n.r.	PPA + MND
	F, 70	n.r.	ALS	PNFA	Language- behavioral + motor	n.r.	+	+	n.a.	−	20 months	Morph.: widespread	n.r.	PPA + MND
	F, 52	n.r.	ALS	PNFA	Behavioral	n.r.	+	+	+	−	24 months	Morph. and funct.: F-T	Aspecific (ND)	n.r.
Soliveri et al., 2003	n.r.	n.r.	ALS	SD	Motor	n.r.	+	+	−	−	n.r.	Morph.: widespread	n.r.	n.r.
	n.r.	n.r.	ALS	PNFA	Motor	n.r.	+	+	−	−	n.r.	Morph.: −	n.r.	n.r.
	n.r.	n.r.	ALS	PNFA	Motor	n.r.	+	+	−	−	n.a.	Morph.: −	n.a.	MND → PPA
Catani et al., 2004	M, 71	n.r.	n.s.	n.s.	Language	n.r.	+	+	−	−	15 months	Morph. and funct.: F-T (L > R)	UP	PPA → MND 6 months
Yokota et al., 2006	M, 55	n.r.	PUMN ALS	SD	Language	R	+	+	−	−	19 years	Morph. and funct.: F-T (L > R)	UP	PPA → MND 16 years
Da Rocha et al., 2007	M, 61	n.r.	ALS	PNFA	Language	n.r.	+	+	n.a.	−	10 months	Morph.: T (L)	UP	PPA + MND
Benajiba et al., 2009	F, 59	*TARDBP*	ALS	SD	Language	n.r.	+	+	−	−	n.r.	Funct.: T	n.r.	PPA → MND 24 months
De Souza et al., 2009	M, 66	n.r.	ALS	SD	Behavioral	R	+	−	−	−	36 months	Funct.:T-P (L > F); F morph.: T	n.r.	PPA → MND 12 months
Kim et al., 2009	M, 61	n.r.	ALS	SD	Behavioral	n.r.	+	+	n.a.	−	n.a.	Morph. and funct: T (R > L)	n.a.	PPA + MND
Espay et al., 2011	F, 70	n.r.	n.s.	PNFA	n.r.	n.r.	+	n.r.	n.r.	Rigidity; oculomotor impairment; imbalance; bradykinesia	14 months	Morph.: F-T	TDP-43	n.r.
	F, 70	n.r.	n.s.	PNFA	n.r.	n.r.	+	n.r.	n.r.	Rigidity; oculomotor impairment; imbalance; bradykinesia	14 months	Morph.: insular	TDP-43	n.r.
Östberg and Bogdanoviæ, 2011	M, 59	−	PLMN ALS	SD	Language	n.r.	+	+	−	−	8 years	Morph.: T (R > L)	TDP-43-B	PPA → MND; 7 years
Coon et al., 2012	F, 72	n.r.	ALS	SD	n.r.	n.r.	+	+	−	−	41 months	Morph.: T (R > L)	TDP-43-B	n.r.
	F, 58	n.r.	ALS	SD	Language-behavioral	L	+	−	−	−	11 months	Morph.: T (R > L)	TDP-43-B	PPA → MND; 6 months
	F, 64	n.r.	PUMN ALS	SD	Motor	L	−	n.a.	n.a.	−	48 months	Morph.: T (R > L) funct.: T (R > L)	n.r.	MND → PPA; 12 months
Pelin et al., 2012	F, 64	n.r.	ALS	PNFA	Language	n.r.	+	+	−	−	n.r.	Morph.: − funct.: F	n.r.	PPA → MND 30 months
Cannon et al., 2013	F, 67	*GRN* (*A9D*)	ALS	n.s.	Motor	n.r.	+	−	+	Rigidity; unbalance; oculomotor impairment; hypomimia	24 months	Morph. and funct.: T (R > L)	TDP-43-B	MND → PPA ≈12 months
Cerami et al., 2013	M, 55	*C9orf72*	ALS	SD	Language	n.r.	+	−	−	−	16 months	Morph.: widespread	n.r.	PPA → MND
Czell et al., 2013	M, 46	*OPTN*	ALS	n.s.	Language	n.r.	+	−	−	−	18 months	Morph.: widespread	n.r.	PPA → MND 6 months
Bäumer et al., 2014	M, 71	n.r.	Mills syndrome	PNFA	Language + motor	R	+	−	−	−	≈24 months	Morph.: T (L)	TDP-43	PPA + MND
Caroppo et al., 2015	n.r., 64	*TBK1*	ALS	SD	Language	n.r.	+	−	−	−	5 years	Morph.: T (L > R)	n.r.	PPA → MND 24 months
	n.r., 68	*TBK1*	ALS	SD	Language	n.r.	−	−	−	Akinetic-rigid parkinsonism	36 months	Morph.: T (L > R); F funct.: T (L > R)	n.r.	PPA → MND 24 months
	n.r., 60	*TBK1*	ALS	PNFA	Language	n.r.	+	+	−	CBS	5 years	Morph.: T (L > R)	n.r.	PPA → MND 48 months
	n.r., 76	*TBK1*	ALS	PNFA	Language	n.r.	−	−	−	−	48 months	Morph. and funct.: F-T (L > R)	n.r.	PPA → MND 24 months
Floris et al., 2015	F, 65	*TARDBP*	ALS	PNFA	Language	n.r.	n.r.	n.r.	n.r.	−	n.a.	Morph. and funct.: F-T (L > R)	n.a.	PPA → MND 5 years
Caroppo et al., 2016	n.r., 59	*TARDBP*	ALS	SD	Language	n.r.	+	+	−	−	7 years	Funct.: T	n.r.	PPA → MND 24 months
	n.r., 71	*TARDBP*	ALS	SD	Language	n.r.	−	n.a.	n.a.	−	7 years	Morph. and funct.: F-T	n.r.	PPA → MND 36 months
Nouini et al., 2017	M, 75	n.r.	ALS	SD	Language	n.r.	+	n.r.	−	−	≈48 months	Morph.: widespread; T (R > L) funct: widespread; T	TDP-43	PPA → MND; ≈36 months
De Marchi et al., 2019	M, 60	−	ALS	PNFA	Language	n.r.	+	+	−	−	37 months	Morph.: widespread, T (L > R) funct.: F, T (L)	n.r.	PPA → MND 22 months
Jiao et al., 2018	F, 63	*TBK1*	ALS	SD	Language	R	+	−	−	−	n.a.	Morph. and funct.: T (L)	n.a.	PPA → MND 12 months
Gazulla et al., 2019	F, 52	−	PLS	PNFA	Motor	n.r.	+	−	−	−	25 years	n.r.	n.r.	MND → PPA
	F, 70	−	PLS	PNFA	Motor	n.r.	+	−	−	−	15 years	n.r.	Aspecific (ND)	MND → PPA 12 years
	M, 55	−	PLS	PNFA	Motor	n.r.	+	−	−	−	20 years	n.r.	n.r.	n.r.
	M, 62	−	PLS	PNFA	Motor	L	+	−	−	Hypomimia; bradikynesia	10 years	Morph: F-P	Aspecific (ND)	MND → PPA 4 years
	M, 58	. −	PLS	PNFA	Motor	n.r.	+	−	−	−	12 years	n.r.	n.r.	MND → PPA
Hirsch-Reinshagen et al. (2019)	M, 68	*TBK1*	PLS	n.s.	Language	R	+	−	−	−	36 months	Morph.: T (L > R) funct: T-P (L > R)	TDP-43-B	PPA → MND
	M, 62	*TBK1*	PLS	PNFA	Language + motor	R	+	−	−	Oculomotor impairment; hypomimia	5 years	Morph.: F-T (L > R)	TDP-43-B	PPA + MND
Rajagopalan and Pioro, 2019, 2021	F, 69	−	ALS	PNFA	Language	n.r.	+	+	+	−	n.a.	Morph. and funct.: F-T (L > R)	n.a.	n.r.
Vonk et al., 2020	F, 63	−	ALS	SD	Language-behavioral	n.r.	+	n.r.	−	−	n.a.	Morph.: F-T (R > L)	n.a.	PPA → MND 9 years
Bergner et al., 2020	F, 61	*FIG4*	PLS	PNFA	Behavioral	n.r.	+	+	n.r.	−	n.a.	Morph: −	n.a.	PPA → MND
Lee et al., 2020	M, 64	n.r.	n.s.	SD	Language	n.r.	+	+	−	Rigidity; oculomotor impairment	7 years	Morph.: T (L)	TDP-43-B	PPA → MND 24 months
	M, 60	n.r.	n.s.	PNFA	Language	n.r.	+	+	−	Rigidity; oculomotor impairment; bradykinesia	5 years	Morph.: widespread	TDP-43-B	PPA → MND 12 months
	M, 51	n.r.	n.s.	PNFA	Language	n.r.	+	+	−	Oculomotor impairment	48 months	Morph.: widespread	TDP-43-B	PPA → MND 36 months
Feng et al., 2021	M, 57	*TARDBP*	ALS	SD	Language	L	−	n.a.	n.a.	−	n.a.	Morph. and funct.: F-T	n.a.	PPA → MND
Li et al., 2021	M, 66	*SQSTM1*	PBP	PNFA	Language	n.r.	+	+	+	−	n.r.	Funct.: F (L > R)	n.r.	PPA → MND
Miki et al., 2020	F, 56	n.r.	PLS	SD	Behavioral	L	−	n.a.	n.a.	−	12 years	morph.: T (R > L)	TDP-43-C	PPA → MND 7 years

ALS, amyotrophic lateral sclerosis; A9D, missense mutation of the progranuline gene; AD, Alzheimer’s disease; bvFTD, behavioral variant frontotemporal dementia; C9orf72, chromosome 9 open reading frame 72; DD, disease duration; EPD, extrapyramidal disorder; F, frontal; FIG4, FIG4 phosphoinositide 5-phosphatase gene mutation; FTD-LBD, Lewy body disease; MND, frontotemporal degeneration with motor neuron disease; funct., functional neuroimaging; GRN, progranulin; MND, motor neuron disease; MNSs, motor neuron signs; morph., morphological neuroimaging; n.a., not applicable; n.r., not retrievable; n.s., not specified; OPTN, optineurin; P, parietal; PBP, progressive bulbar palsy; PNFA, progressive non-fluent aphasia; PPA, primary progressive aphasia; PLMN, predominant lower motor neuron; PLS, primary lateral sclerosis; PUMN, predominant upper motor neuron; SD, semantic dementia; SQSTM1, sequestosome 1; T, temporal; TARDBP, TAR DNA-binding protein; TBK1, TANK binding kinase 1; TDP-43, TAR DNA-binding protein (letters following the acronym represents the type sensu Mackenzie (Mackenzie et al., 2011); UP, ubiquitin-positive inclusions.

**TABLE 2:** Group studies.

Author, year	Sampling	Condition (diagnosis), (*N*)	PPA phenotype,% (*N*)	MND/MN dysfunction,% (*N*)	Main histological findings % (*N*)	Sex (M/F), age (year)	Survival (months)	Bulbar signs	EPD	Genetics,% (*N*)	Clinical course, latency
Caselli et al., 1993	Prospective (longitudinal); clinic-based	ALS-FTD/FTD-MND (clinical), *N* = 7	n.s.: +	ALS	n.r.	3/4; *M* = 67	n.r.	+	−	n.r.	n.r.
Rakowicz and Hodges, 1998	Prospective (cross-sectional); population-based	ALS (clinical), *N* = 18	n.s.: 27.8% (5)	n.a.	n.r.	3/2, *M* = 65	n.a.	80% (4)	−	n.r.	MND → PPA: 40% (2) PPA → MND: 20% (1)^1^
Hamilton and Bowser, 2004	Retrospective; clinic-based	ALS^2^ (clinical), *N* = 29	n.s.: 17.2% (5)	n.a.	UP: 60% (3)	4/1, *M* = 69	*M* = 32	n.r.	n.r.	n.r.	n.a.
Davies et al., 2005	Retrospective; clinic-based	SD (clinical), *N* = 18	n.a.	MN dysfunction: 9% (2)	UP	2/0, 61/73	234/120	50% (1)	−	n.r.	PPA → MND
Kertesz et al., 2005	Retrospective (longitudinal); clinic-based	PPA (clinical), *N* = 22	n.s.	−	UP: 22.2% (4)	n.r.	n.r.	n.r.	CBS: 50% (2)	n.r.	n.a.
		PPA + bvFTD (clinical), *N* = 20	n.s.	MND: 15% (3)	UP	n.r.	n.r.	n.r.	−	n.r.	PPA → MND: 2.3 years
		SD + bvFTD (clinical), *N* = 2	n.a.	−	UP: 50% (1)	n.r.	n.r.	n.r.	−	n.r.	n.a.
Pickering-Brown et al., 2008	Prospective (cross-sectional); clinic-based	FTD, *N* = 223	PNFA: 9% (20)	MN dysfunction: 15.4% (2)	n.r.	n.r.	n.r.	n.r.	n.r.	n.r.	n.a.
			SD: 13% (29)	−	n.r.	n.r.	n.r.	n.r.	n.r.	n.r.	n.a.
			PNFA + bvFTD: 4% (9)	−	n.r.	n.r.	n.r.	n.r.	n.r.	n.r.	n.a.
			SD + bvFTD: 19% (43)	MN dysfunction: 16.3% (7)	n.r.	n.r.	n.r.	n.r.	n.r.	n.r.	n.a.
Kobayashi et al., 2010	Retrospective (longitudinal); clinic-based	FTD-MND (histological), *N* = 16	SD: 37.5% (6)	PUMN ALS: 83.3% (5) ALS: 16.7% (1)	TDP-43-A: 16.7% (1) TDP-43-B: 16.7%% (1) TDP-43-C: 66.7% (4)	4/2, *M* = 53.3	*M* = 36	−	33.3% (2)	n.r.	PPA → MND
			n.s.: 6.3% (1)	PUMN ALS	TDP-43-B	M, 75	69.6	−	−	n.r.	n.a.
Burrell et al., 2011	Prospective (cross-sectional); clinic-based	PPA (clinical), *N* = 22	PNFA: 54.5% (12)	MND: 16.7% (2) MN dysfunction: 33.3% (4)	n.r.	n.a.	n.a.	+	−	n.r.	n.a.
			SD: 45.5% (10)	MN dysfunction: 20% (2)	n.r.	n.a.	n.a.	+	−	n.r.	n.a.
Hsiung et al., 2012	Prospective (longitudinal); clinic-based	ALS-FTD (clinical), *N* = 7	PNFA + bvFTD: 71.4% (5) PNFA: 14.3% (1)	n.a.	TDP-43-B	3/3, *M* = 51	*M* = 31.2	+	66.7% (4)	*C9orf72*	PPA → MND: 60% (3); 1.6 years PPA + MND: 20% (1) MND → PPA: 20% (1); 1 year
		PNFA + bvFTD (clinical), *N* = 2	n.a.	MN dysfunction	TDP-43-B: 50% (1) TDP-43-A + B: 50% (1)	M, *M* = 59	*M* = 114	50% (1)	50% (1)	*C9orf72*	n.r.
Tremolizzo et al., 2014	Prospective (cross-sectional); clinic-based	PPA (clinical), *N* = 12	n.s.	MN dysfunction: 16.7% (2)	n.r.	n.r.	n.r.	n.r.	n.r.	n.r.	n.a.
Dols-Icardo et al., 2015	Prospective (cross-sectional); population-based	FTD-MND (clinical), *N* = 92	PNFA: 6.6% (6) SD: 1.3% (2)	n.a.	n.r.	n.r.	n.r.	n.r.	n.r.	−	n.a.
Matias-Guiu et al., 2015	Prospective (longitudinal); clinic-based	PPA (clinical), *N* = 35	PNFA: 34.3% (12)	ALS: 25% (3)	n.r.	5/7, *M* = 75.5	n.a.	n.r.	n.r.	−	PPA → MND 2 years
			SD: 11.4% (4)	−	n.r.	2/2, *M* = 66.5	n.a.	n.r.	n.r.	n.a.	n.a.
			lvPPA: 48.6% (17)	−	n.r.	6/11, *M* = 76.7	n.a.	n.r.	n.r.	n.a.	n.a.
			n.s.: 5.7% (2)	−	n.r.	1/1, *M* = 77	n.a.	n.r.	n.r.	n.a.	n.a.
Saxon et al., 2017a	Retrospective; clinic-based	ALS-FTD (clinical), *N* = 89	PNFA: 1.1% (1) SD: 3.4% (3) SD + bvFTD: 11.2% (10) PNFA + bvFTD: 5.6% (5)	n.a.	n.r.	n.r.	n.r.	n.r.	n.r.	n.r.	n.r.
Van Langenhove et al., 2017	Prospective (longitudinal); clinic-based	PPA ± bvFTD (clinical), *N* = 86	PNFA: 34.9% (30)	ALS: 3.3% (1)	n.r.	n.r.	n.r.	−	n.r.	n.r.	PPA → MND: < 54 months
			PNFA + bvFTD: 12.8% (11)	ALS: 45.5% (5)	n.r.	n.r.	n.r.	+	n.r.	n.r.	
			SD: 27.9% (24)	−	n.r.	n.r.	n.r.	n.r.	n.r.	n.r.	n.a.
			SD + bvFTD: 24.4% (21)	−	n.r.	n.r.	n.r.	n.r.	n.r.	n.r.	
Kartanou et al., 2018	Prospective (cross-sectional); clinic-based	PPA (clinical), *N* = 18	PNFA: 66.7% (12)	ALS: 8.3% (1)	n.r.	F, 45	n.r.	n.r.	n.r.	*C9orf72*	n.a.
			SD: 32.3% (6)	−	n.r.	n.r.	n.r.	n.r.	n.r.	−	n.a.
Perry et al., 2019	Retrospective (longitudinal); clinic-based	PPA (clinical), *N* = 91	PNFA: 35.2% (32)	ALS: 3.1% (1)	n.r.	n.r.	n.r.	n.r.	n.r.	n.r.	PPA → MND
			SD: 74.8% (59)	MND (n.s.): 1.7% (1)	n.r.	n.r.	n.r.	n.r.	n.r.	n.r.	n.r.
Tan et al., 2019	Prospective (cross-sectional); clinic-based	PPA (clinical), *N* = 130	PNFA: 51% (66)	ALS: 18% (12)	TDP-43-A 14.3%^3^ (1) TDP-43-B 42.9%^3^ (3) TDP-43-E 14.3%^3^ (1)	4/8, *M* = 63.5	*M* = 28	+	−	*C9orf72*: 33.3% (4)	n.a.
			SD: 49% (64)	ALS: 5% (3)	TDP-43-B 66.6% (2) TDP-43-E 33.3% (1)	1/2, *M* = 62	*M* = 12	+	−	−	n.a.
Vinceti et al., 2019	Retrospective; clinic-based	FTD-MND (histological), *N* = 32	PNFA: 9.4% (3)	n.a.	TDP-43-A: 33.3% (1) TDP-43-B: 66.6% (2)	2/1, *M* = 55	*M* = 45.6	+	n.r.	n.r.^4^	PPA + MND
			SD: 21.9% (7)	n.a.	TDP-43-A: 14.3% (1) TDP-43-B: 57.1% (4) TDP-43-C: 28.6% (2)	5/2, *M* = 57.3	*M* = 139.2	+	n.r.	n.r.	PPA + MND: 57.1% (4) PPA → MND: 28.6% (2); 9.8 years ^1^
Crespi et al., 2020	Prospective (cross-sectional); clinic-based	PPA (clinical), *N* = 16	PNFA: 62.5% (10)	MN dysfunction: 67% (4)	n.r.	n.r.	n.r.	n.r.	n.r.	n.r.	n.a.
			SD: 37.5% (6)	MN dysfunction: 33% (2)	n.r.	n.r.	n.r.	n.r.	n.r.	n.r.	n.a.
Ulugut et al., 2021	Retrospective (longitudinal); clinic-based	PPA (clinical), *N* = 64	PNFA: 34.4% (22)	MN dysfunction: 4.5% (1)	n.r.	n.r.	n.r.	n.r.	−	*C9orf72*	PPA → MND: 1 year
			SD: 37.5% (24)	−	n.r.	16/8, *M* = 63.6	*M* = 43	n.r.	n.a.	n.a.	n.a.
			lvPPA: 28.1% (18)	−	n.r.	8/10, *M* = 66.5	*M* = 40	n.r.	n.a.	n.a.	n.a.

ALS, amyotrophic lateral sclerosis; bvFTD, behavioral variant frontotemporal dementia; C9orf72, chromosome 9 open reading frame 72; DD, disease duration; EPD, extrapyramidal disorder; FTD-MND, frontotemporal degeneration with motor neuron disease; lvPPA, logopenic variant primary progressive aphasia; MND, motor neuron disease; MN, motor neuron;, months; n.a., not applicable; n.r., not retrievable; n.s., not specified; PNFA, progressive non-fluent aphasia; PPA, primary progressive aphasia; PLMN, predominant lower motor neuron; PUMN, predominant upper motor neuron; SD, semantic dementia; TDP-43, TAR DNA-binding protein (letters following the acronym represents the type sensu Mackenzie (Mackenzie et al., 2011); UP, ubiquitin-positive;. ^1^Onset type-related information not retrievable for certain patients; ^2^One case showed PPA before motor signs; ^3^Percentages computed on the number of PNFA patients with available neuropathology (N = 7); ^4^One patient showed C9orf72 hexanucleotide repeat expansion, although it was not specified whether within the PNFA or SD group.

### Case reports and series

Within case reports and case series, 61 patients were described with PPA-MND (55.8% males; mean age: 62 ± 7.5 years, *range* = 43–76). Onset symptoms distributed as follows: only language: 50.9%; only motor: 17.5%; only behavioral: 10.5% language + motor: 10.5%; language + behavioral: 7%; motor + behavioral ± language: 3.5%. The median delay between the occurrence of PPA and MND/MN dysfunction was 24 months (mean: 40.5 ± 44), whereas that between MND/MN dysfunction and PPA was 12 months (mean: 24 ± 20.6), with 65.4% of patients showing PPA first. PNFA was more prevalent (60.8%) than SD (39.2%). Classical amyotrophic lateral sclerosis (ALS) was the most prevalent MND phenotype (70.2%), followed by predominant-UMN phenotypes (25.5%), including primary lateral sclerosis and Mills’ syndrome (Jaiser et al., 2019). The lifetime prevalence of bulbar signs was 88.3%, whilst that at MND onset was 56%. In 15.2% of “PPA first” patients, bulbar involvement preceded that of other bodily regions. The lifetime prevalence of extrapyramidal involvement was 16.4%. Survival was highly heterogeneous, with a median of 36.5 months (mean: 62.2 ± 64.6 months), ranging from 10 months to 25 years. Neuroradiological data were overall consistent with a left-predominant, frontotemporal, perisylvian involvement, except for 14% of patients showing SD with a selective right-sided temporal involvement. The most frequent *post-mortem* finding was, for studies before 2007 (*N* = 9 patients with available neuropathology), ubiquitin-positive inclusions (66.7%), while, for studies after 2007 (*N* = 17 patients with available neuropathology), TDP-43-B pathology [52.9%; *sensu* Mackenzie (Mackenzie et al., 2011)]. Among genetic-positive patients (*N* = 17), the most frequently mutated gene was *TBK1* (41.2%), followed by *TARDBP* (29.4%).

### Group studies

Group studies reporting PPA-MND patients addressed heterogeneous conditions falling under the FTD (*N* = 12), ALS-FTD (i.e., ALS as the primary diagnosis; *N* = 2) or FTD-MND (i.e., FTD as the primary diagnosis; *N* = 3) phenotypes (Strong et al., 2017) – except for two, which addressed ALS (Rakowicz and Hodges, 1998; Hamilton and Bowser, 2004), and another not specifying which the primary diagnosis was (either ALS-FTD or FTD-MND) (Caselli et al., 1993). Longitudinal analyses were detected in eight studies, focused either on PPA (*N* = 5) or ALS-FTD/FTD-MND (*N* = 3).

Seven eligible studies addressing PPA patients were entered into the proportional meta-analysis, yielding an estimate of lifetime prevalence of MND/MN dysfunction in PPA of 6% (CI 95% [3%, 9%]) (Supplementary Figure 2). When separately assessing PNFA and SD (*N* = 4 eligible studies), such a prevalence was of 10% in PNFA (CI 95% [3%, 17%]) and of 3% in SD (CI 95% [0%, 6%]) (Supplementary Figure 3). MND/MN dysfunction was never reported in lvPPA patients within the two studies also addressing this phenotype (Matias-Guiu et al., 2015; Ulugut et al., 2021).

As to the prevalence of PPA in ALS-FTD/FTD-MND, the 3 eligible, entered studies yielded an estimate of 19% (CI 95% [6%, 31%]) (Supplementary Figure 4) – and, when separately assessing PNFA and SD, of 7% for the former CI 95% [4%, 10%]) and of 11% for the latter (CI 95% [0%, 23%]), respectively (Supplementary Figure 5).

Besides prevalence- and phenotype-related information on PPA phenotypes, remaining data was often non-retrievable for individual patients, and thus could not be qualitatively summarized (Table 1). However, regarding neuropathology, it is worth mentioning that the large study by Tan et al. (2019), which addressed 130 PPA patients, of whom 11.5% had concurrent ALS, converged with case reports/series as to TDP-43-B being the most prevalent pathological substrate (50%). Moreover, with respect to genetics, it has to be noted that, within the same study, the sole, incidental finding in mutated patients (showing PNFA) was *C9orf72* hexanucleotide repeat expansion (HRE) (Tan et al., 2019).

## Discussion

The present study summarizes current evidence on the association between PPA and MND/MN dysfunction (i.e., PPA-MND) at clinical, pathological and genetic levels (Burrell et al., 2016).

### Clinical phenotypes

The lifetime prevalence of MND/MN dysfunction in PPA patients was of 6%. Although no meta-analytic evidence on the topic is available for bvFTD, empirical studies suggest that, in such a phenotype, this prevalence would be similar (Cerami et al., 2015), or slightly higher (Tremolizzo et al., 2014; Ganapathy et al., 2020). Moreover, when separately addressing PPA phenotypes, such an estimate was higher for PNFA (10%) than for SD (3%) – this being explainable according to a corticofugal model of TDP-43-mediated neurodegeneration: indeed, PNFA primarily affects frontal-insular areas contiguous to motor regions, whereas SD mostly involves anterior temporal lobes, which are topographically distant from motor cortices (Braak et al., 2013; Brettschneider et al., 2013).

By contrast, the finding of MND/MN dysfunction never co-occurring with lvPPA was in line with the latter being commonly underpinned by a non-FTLD neuropathology (Roytman and Chiang, 2022).

The finding of 19% of ALS-FTD/FTD-MND patients presenting with PPA (with similar estimates for PNFA and SD – i.e., 7 and 11%, respectively) was likewise expected, given that PPA is known to be less commonly associated to MND than bvFTD (Saxon et al., 2017a,b; Lulé et al., 2019).

As to MND phenotypes, regardless of PPA variants, classical ALS was the most prevalent (70.2%), followed by predominant-UMN phenotypes (25.5%) – with only one patient (2.1%) showing a predominant-LMN phenotype. As to patients primarily diagnosed with FTD, such findings are unsurprising: indeed, it is unlikely that, in the presence of such a cortical pathology, UMNs are less involved than LMNs (McKenna et al., 2021). Conversely, as to primarily MND patients, these results align with recent evidence on extra-motor cortical burden being greater when UMNs are involved (Poletti et al., 2021; Sbrollini et al., 2021; Aiello et al., 2022; Maranzano et al., 2022).

The high lifetime prevalence of bulbar signs (88.3%), as well as the moderate proportion of bulbar-onset MNDs (56%), is then consistent with bulbar involvement being acknowledged, within the MND-FTD *spectrum*, as a risk factor for cognitive deficits (Yang et al., 2021), as well as with it being specifically linked to impairments of language as compared to other cognitive domains (Shellikeri et al., 2017).

As to extrapyramidal signs/syndromes, the present prevalence in PPA-MND (16.4%) overall resembles that previously detected in bvFTD (22.7%) (Padovani et al., 2007) and MND alone – ranging from 1.7 to 15.7% (McCluskey et al., 2014; Pupillo et al., 2015; Pasquini et al., 2022), being nevertheless lower than current estimates addressed to PPAs (i.e., up to 40%) (Kremen et al., 2011; Armstrong et al., 2013; Höglinger et al., 2017; Ulugut et al., 2021).

A relatively stable finding was patients’ age at onset/clinical referral, being among the fourth and seventh decade (62 years on average) – this overall aligning with the current epidemiological knowledge on PPA (Montembeault et al., 2018) and MND (Longinetti and Fang, 2019).

As to the median survival of PPA-MND patients – i.e., ≈3 years –, it was slightly lower than that of patients with PPA only (Montembeault et al., 2018), this agreeing with the fact that MN involvement represents a risk factor for a shorter survival in PPA patients (De La Sablonnière et al., 2021; El-Wahsh et al., 2021). Its high heterogeneity herewith found might be accounted for by the diversities across both PPA and MND phenotypes – since survival is longer in SD than in PNFA (Tastevin et al., 2021), and shorter in classical ALS as compared to atypical MND variants – such as predominant-UMN phenotypes (Turner and Talbot, 2020), which were relatively highly represented (25.5%) among PPA-MND patients.

Finally, PPA-MND patients frequently appeared (65.4%) to present with PPA first. However, a considerable, and extremely variable, delay between the two diseases was found – being higher for “PPA-first” patients (median: 24 months) than for “MND-first” ones (median: 12 months). Such findings partially align with a recent investigation (Gromicho et al., 2021) reporting a median delay of 1 year between the onset of bvFTD and MN dysfunction in FTD-MND patients.

### Neuropathology

When referring to post-2007 studies reporting *post-mortem* data according to current TDP-43 classification systems (Mackenzie et al., 2011), the most common histological finding, regardless of PPA variants, was TDP-43-B (≈50% of cases), in line with this subtype being the pathological substrate of ALS-FTD/FTD-MND phenotypes (Mackenzie et al., 2011). However, group studies also showed a considerable heterogeneity of neuropathological features – including TDP-43-A and TDP-43-C, typical of PNFA and SD alone (Mackenzie et al., 2011), respectively, and the and the recently acknowledged TDP-43-E (Lee et al., 2017) – although not allowing inferences on these other substrates due to incomplete data or small sample sizes. Overall, it is likely that, when PPA co-occurs with MND, a one-to-one association between pathology and phenotype might not occur.

### Genetics

*TBK1* was the most frequently mutated gene in genetic-positive PPA-MND patients (41.2%), in accordance with evidence suggesting that loss of function mutations in this gene display a broad phenotypic heterogeneity - encompassing bvFTD, PPA (both PNFA and SD) (Le Ber et al., 2015; Lamb et al., 2019; Swift et al., 2021), MND (and, especially, predominant-UMN phenotypes) (Van Mossevelde et al., 2016; Gómez-Tortosa et al., 2017) and atypical parkinsonisms (Wilke et al., 2018; Seibert et al., 2021; Swift et al., 2021).

Also C-terminal missense mutations in the *TARDBP* gene were detected as a relatively frequent genetic underpinning of PPA-MND (29.4%), although with a disproportion toward SD (80%; PNFA: 20%) – this last finding being in line with several reports (Gelpi et al., 2014; González-Sánchez et al., 2018), also showing that SD is overrepresented in *TARDBP* carriers compared to other FTD phenotypes (Caroppo et al., 2016; van Rooij et al., 2020).

Conversely, *C9orf72* HRE, which represents the major genetic determinant underlying the MND-FTD *spectrum*, appeared not to be ubiquitous – despite a large group study identifying it as the sole genetic feature of mutated PPA-MND patients showing PNFA (Tan et al., 2019). This aligns with evidence suggesting that *C9orf72* HRE carriers are more represented among bvFTD – with or without comorbid MND – than PPA patients (Le Ber et al., 2013; Costa et al., 2020), as well as that this genetic form accounts mostly accounts for PNFA (Saracino et al., 2021).

Lastly, mutations in other genes, namely *GNR*, *OPTN*, *FIG4*, and *SQSTM1*, appeared to be extremely rare or incidental findings in PPA-MND.

### Limitations

A number of limitations have to be acknowledged.

First, only two studies, biased by a small sample size, focused on the prevalence of PPA in MND patients – this preventing this study from providing generalizable estimates of the lifetime prevalence of PPA in MND patients.

Second, several outcomes proved to be irretrievable from group studies, due to the fact that the vast majority of them did not selectively focus on the conjunction between PPA and MND – and that, therefore, findings of interest were mostly incidental. Such a lack is the most obvious for neuroradiological data, which were indeed extracted for case reports/series only. Similarly, although to a lesser extent, data extracted from case reports/series also happened to be incomplete. Relatedly, this study lacks a formal quality assessment, which was not performed due to the fact itself that both case reports/series and group studies infrequently focused on the primary outcome of the present study – i.e., the conjunction between PPA and MND.

Third, the meta-analytic procedures performed within the present study included a very small number of studies, which, moreover, were markedly different from one another as to their design (i.e., cross-sectional vs. longitudinal, prospective *vs.* retrospective) and sample sizes. Such a diversity also prevented from reporting interpretable publication bias statistics. Thereupon, meta-analytic findings herewith reported must be approached with extreme caution, and respective estimates have to be interpreted as gross approximations of underlying parameters.

## Author contributions

ENA: conceptualization, data synthesis, drafting, and revision. SF: conceptualization, data collection, data synthesis, drafting, and revision. GD, LG, and EA: data collection, drafting, and revision. IA, FS, LC, BP, FV, and VS: drafting, revision, and resources. NT: conceptualization, drafting, revision, and resources. All authors contributed to the article and approved the submitted version.

## Footnotes

1
https://rayyan.qcri.org/welcome
