# Supplementary material for: Primary progressive aphasia and motor neuron disease: A review
Source: Front Aging Neurosci. 2022 Sep 8;14:1003792. doi: 10.3389/fnagi.2022.1003792 (PMC9492890; doi:10.3389/fnagi.2022.1003792)
Supplement: Supplementary file 1 [file Data_Sheet_1.docx]

**Supplementary Figure 1.** Study selection process according to PRISMA guidelines.

Duplicates removed

*(N*=242 deleted; 6 not duplicates; 227 resolved)

Records identified through database search
(*N*=788)

## Identification

Records excluded
(*N*=481)

- review, meta-analysis, editorial, commentaries, chapter, conference (*N*=252)

- no PPA (*N*=72)

- no MND (*N*=65)

- no PPA & MND conjunction (*N*=73)

- no patients (*N*=5)

- language (*N*=14)

Records after duplicates removal
(*N*=546)

## Screening

Eligible records identified through reference search
(*N*=9)

Full-text articles assessed for eligibility
(*N*=74)

## Eligibility

Full-text articles excluded
(*N*=18)

- no full-text (*N*=3)

- language (*N*=6)

- insufficient data (only mentioning PPA & MND conjunction; *N*=3)

- no PPA & MND conjunction (*N*=4)

- data overlap (*N*=2)

Studies included in the review
(*N*=56)

## Included

**Notes.** Study selection process according to PRISMA guidelines [Page *et al.*¸2020]

**Supplementary Figure 2.** Forest plot for the estimate of the lifetime prevalence of MND/MN dysfunction in PPA patients.
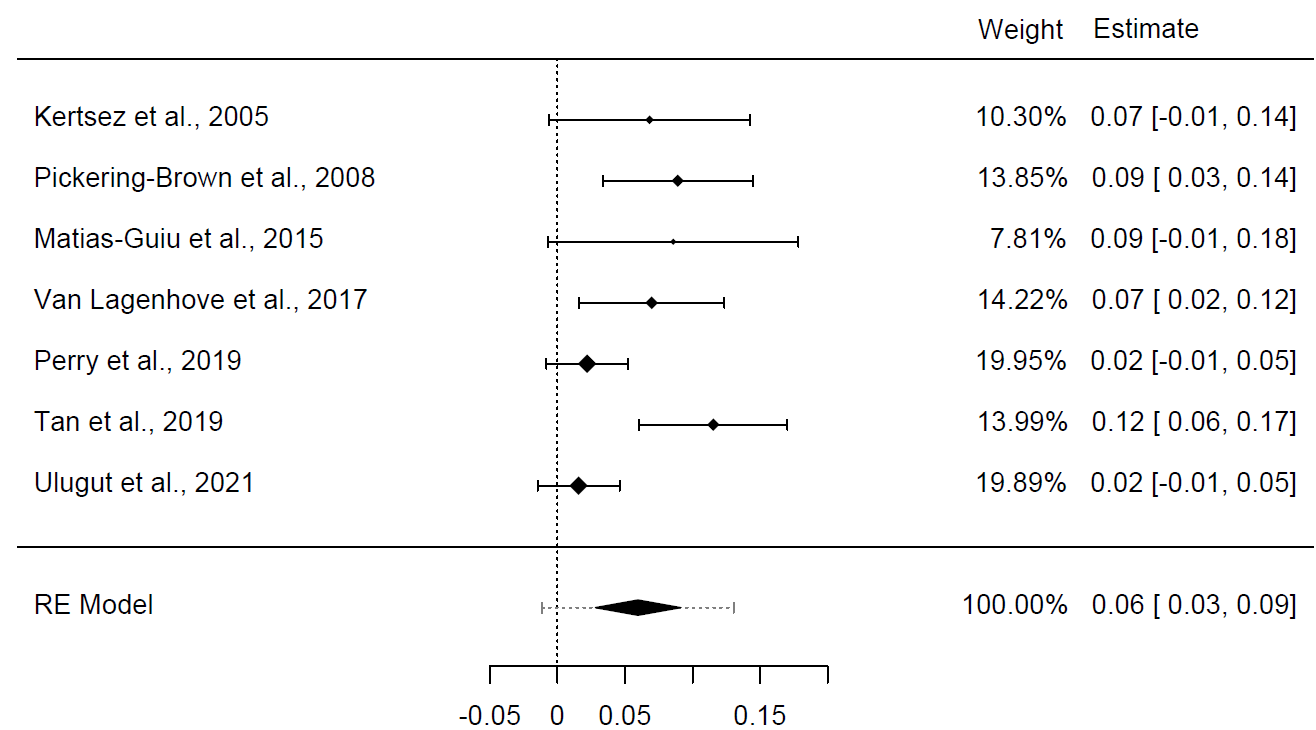


**Notes.** I^2^=64.41%. MND=motor neuron disease; MN=motor neuron; PPA=primary progressive aphasia; RE=random effect.

**Supplementary Figure 3.** Forest plot for the estimate of the lifetime prevalence of MND/MN dysfunction in PNFA and SD patients.


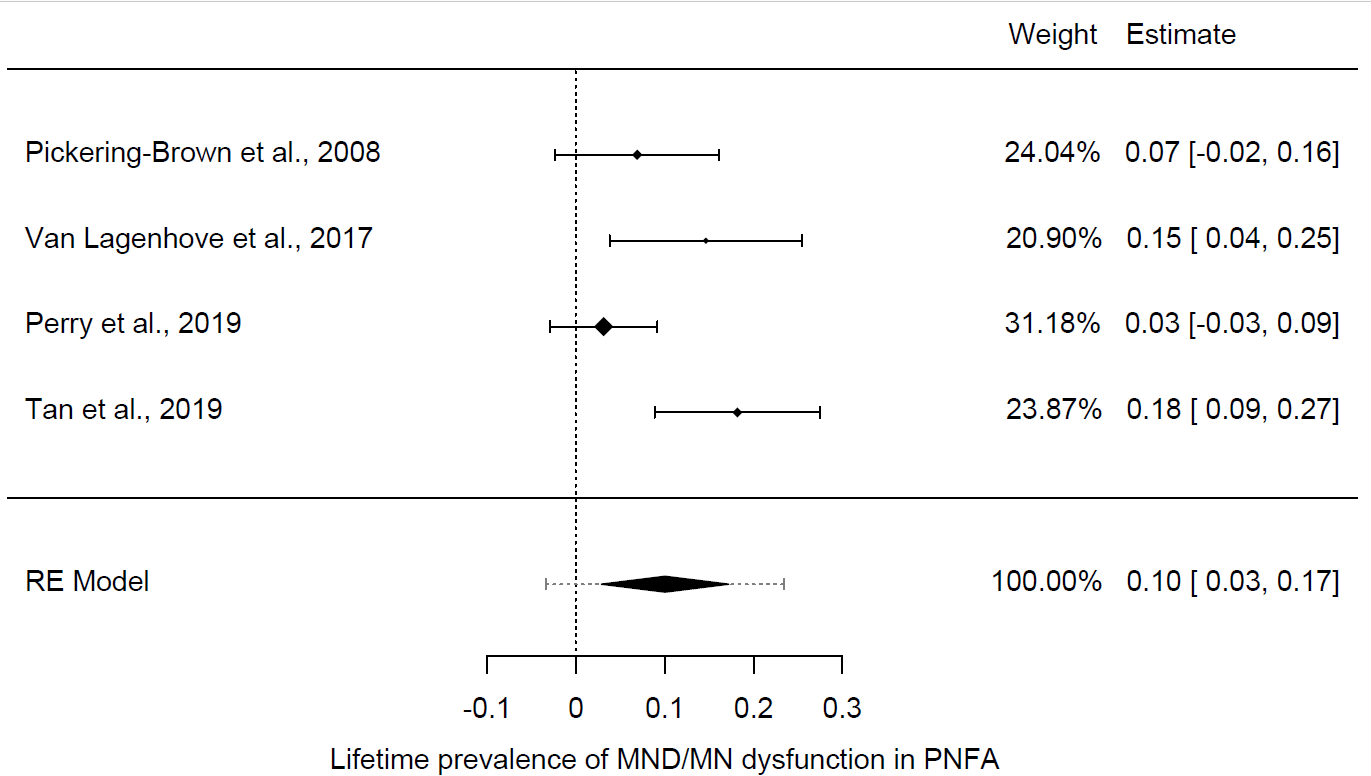

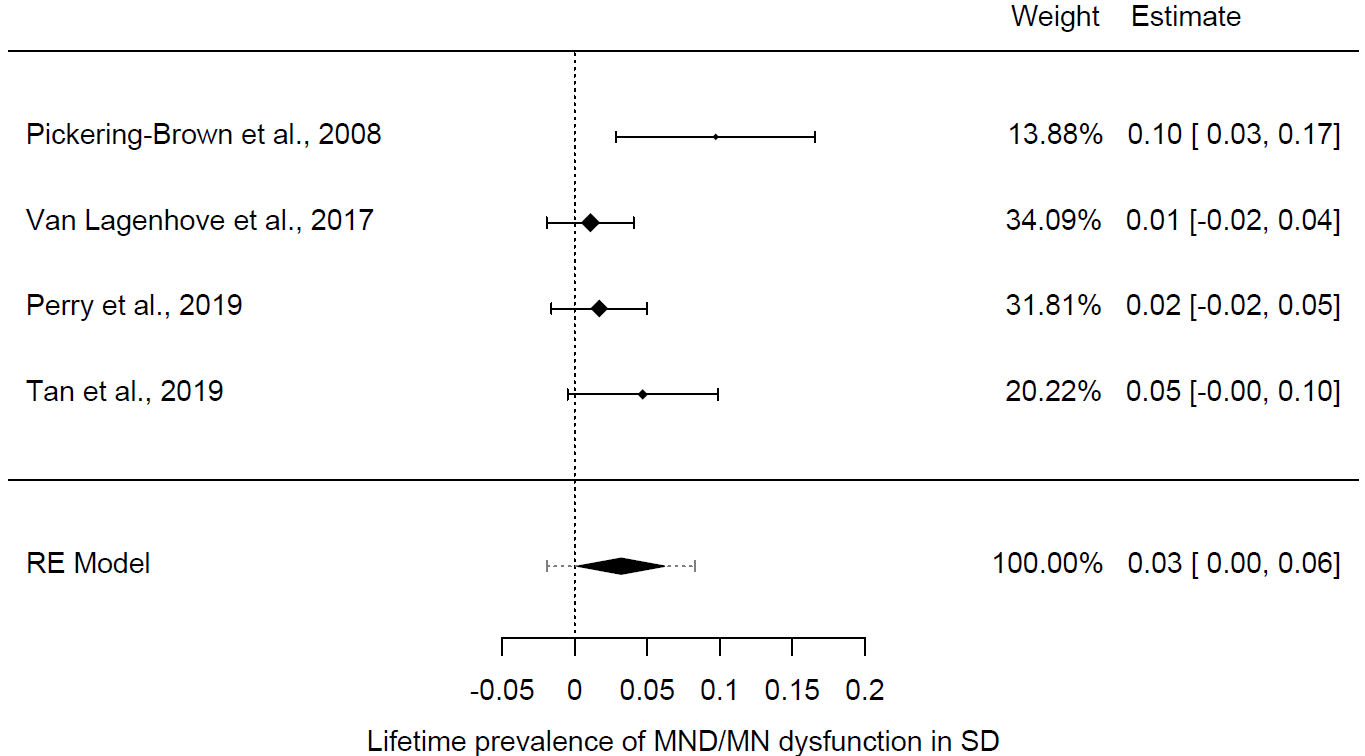


**Notes.** PNFA: I^2^=63.4%; SD: I^2^=49.9%. MND=motor neuron disease; MN=motor neuron; PNFA=progressive non-fluent aphasia; RE=random effect; SD=semantic dementia.

**Supplementary Figure 4.** Forest plot for the estimate of the prevalence of PPA in ALS-FTD/FTD-MND patients.


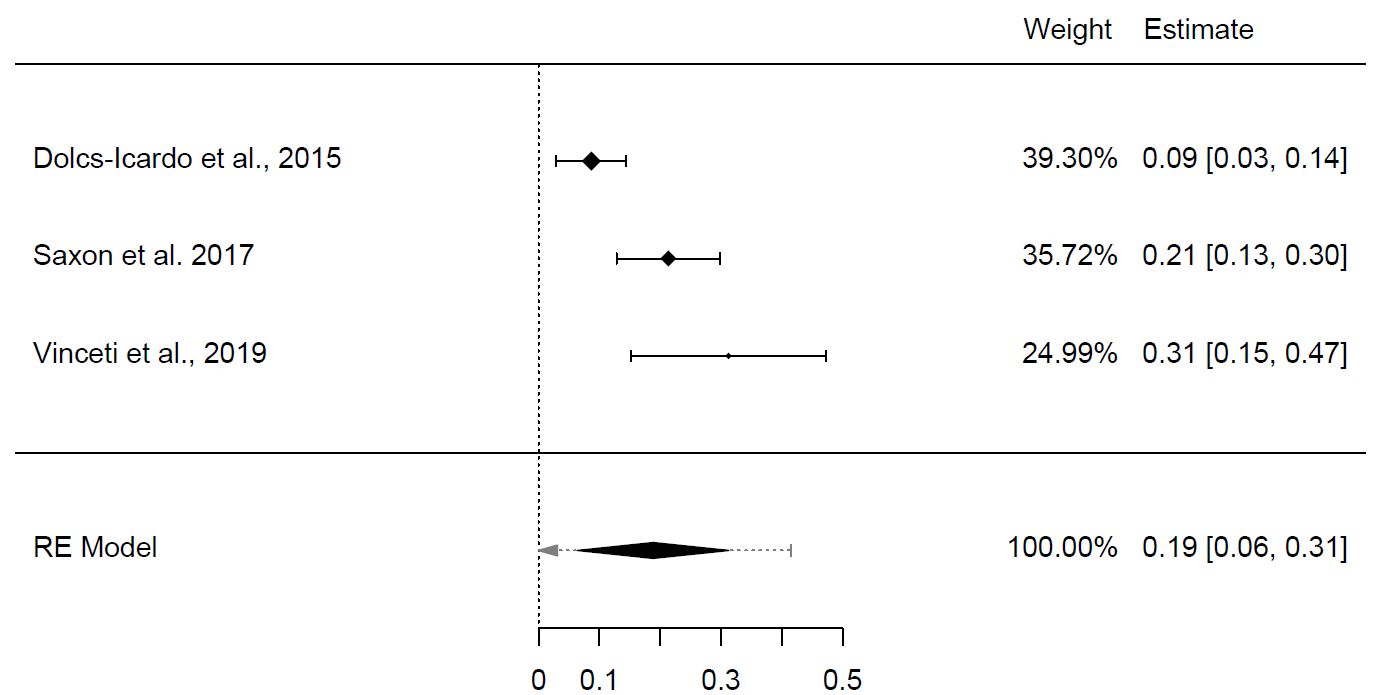


**Notes.** I^2^=81.5%. ALS-FTD=amyotrophic lateral sclerosis with frontotemporal degeneration; FTD-MND=frontotemporal degeneration with motor neuron disease; PPA=primary progressive aphasia; RE=random effect.

**Supplementary Figure 5.** Forest plot for the estimate of the prevalence of PNFA and SD in ALS-FTD/FTD-MND patients.


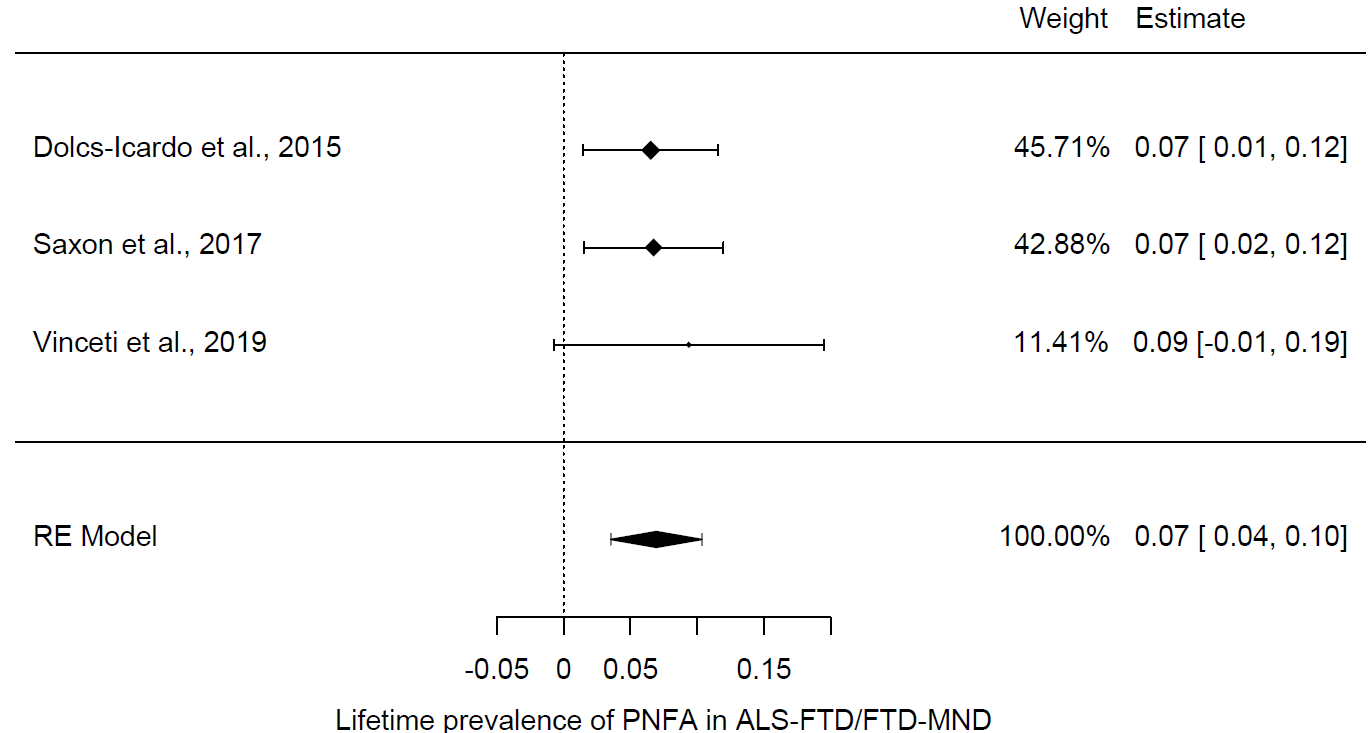


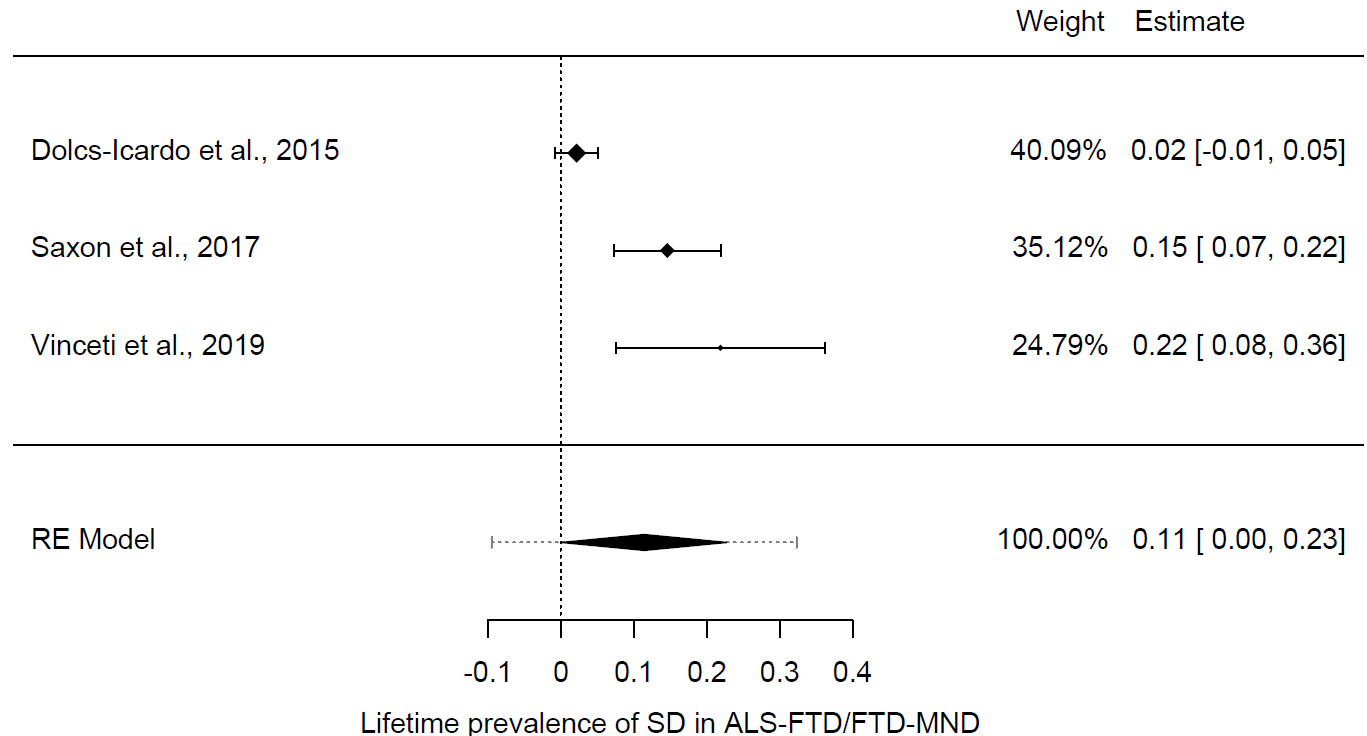


**Notes.** PNFA: I^2^=not estimated; SD: I^2^=86.1%. ALS-FTD=amyotrophic lateral sclerosis with frontotemporal degeneration; FTD-MND=frontotemporal degeneration with motor neuron disease; PNFA=progressive non-fluent aphasia; RE=random effect; SD=semantic dementia.
